# A very rare case of spontaneous regression of basaloid squamous cell carcinoma of the lung

**DOI:** 10.1111/1759-7714.15217

**Published:** 2024-01-13

**Authors:** Sachie Koike, Takayuki Shiina, Keiichiro Takasuna, Akane Kato, Takuma Atagi, Toshitsugu Nakamura

**Affiliations:** ^1^ Department of Thoracic Surgery Ina Central Hospital Nagano Japan; ^2^ Division of General Thoracic Surgery, Department of Surgery Shinshu University School of Medicine Nagano Japan; ^3^ Department of Respirology Ina Central Hospital Nagano Japan; ^4^ Department of Pathology Ina Central Hospital Nagano Japan

**Keywords:** basaloid squamous cell carcinoma of the lung, immune stimulation, lung cancer, lymphocytes, spontaneous regression

## Abstract

Spontaneous regression of non‐small cell lung cancer is relatively rare. Here, we present a very rare case of spontaneous regression of lung cancer which occurred in a patient with basaloid squamous cell lung cancer. To the best of our knowledge, this is the first report of such a case. A 76‐year old man was referred to our hospital with nodules in the right upper lobe determined by chest computed tomography. The nodules spontaneously regressed during follow‐up. Two years later, the tumor had regrown and the patient subsequently underwent surgery. The pathological findings showed basaloid squamous cell carcinoma. Stimulation of the immune system was considered to be the cause of the spontaneous regression and CD‐8 positive and CD‐4 positive lymphocytes might play an important role.

## Graphical abstract

Here, we report a case of spontaneous regression of basaloid squamous cell carcinoma of the lung. To the best of our knowledge, spontaneous regression of basaloid squamous cell carcinoma of the lung has not previously been reported.

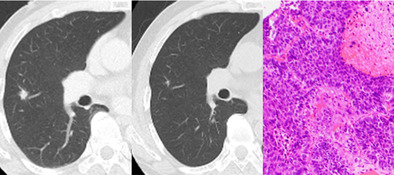

## INTRODUCTION

Spontaneous regression (SR) of cancer is defined as the partial or complete disappearance of primary or metastatic tumor tissue in the absence of treatment.^1^ It occurs approximately once in every 60 000–100 000 cancer cases and most reported cases are melanoma, or hematological primaries, and are commonly attributed to the immune system.^2^ Lung cancers undergo SR less frequently.^2^ Herein, we present a very rare case of SR of basaloid squamous cell carcinoma of the lung, which to the best of our knowledge has not previously been reported in the literature.

## CASE REPORT

**FIGURE 1:**
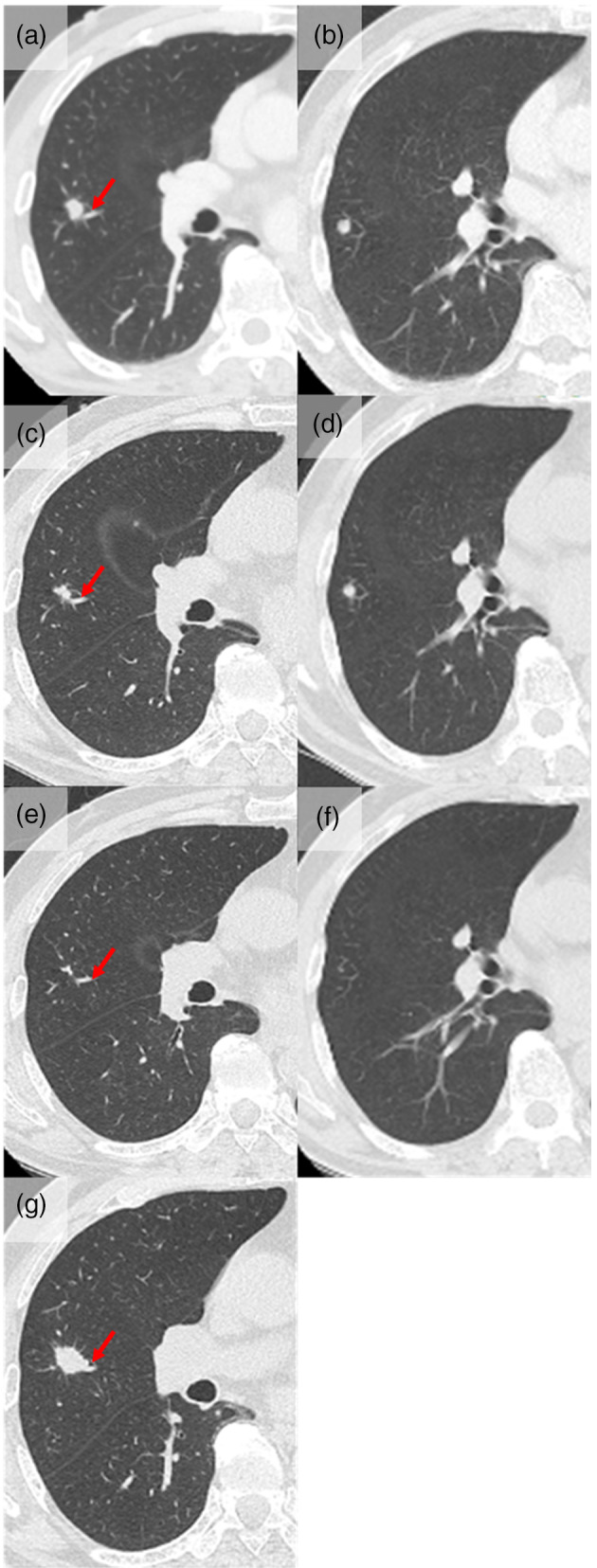
Chest computed tomography (CT) of the tumor. (a) CT revealed a 10.5 x 9.7 mm nodule located in the peripheral section of B2b, close to V3aii (red arrow), and (b) a 7.8 x 5.4 mm nodule located in S2 at the first visit. (c) One month later, the larger nodule had shrunk to 8.1 x 8.4 mm. (d) The size of another nodule had not changed. (e, f) Four months later, both nodules had shrunk to an almost unrecognizable size. (a, g) Two years later, CT revealed a 19.3 x 10.4 mm nodule located in the peripheral section of B2b, close to V3aii (red arrow), in exactly the same place as the larger nodule revealed 2 years previously.

**FIGURE 2:**
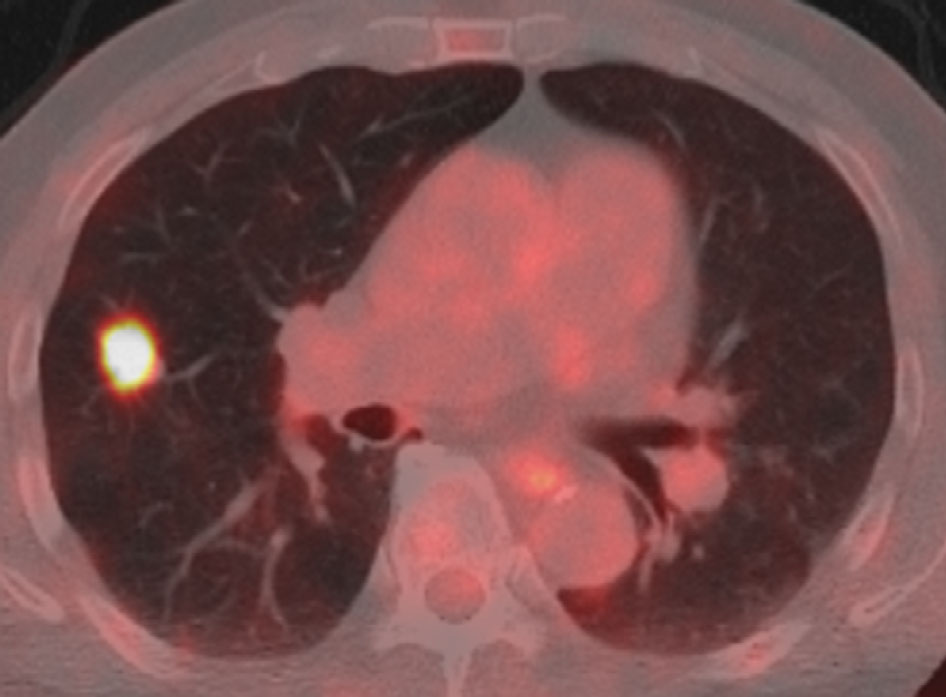
Positron emission tomography/computed tomography (PET/CT) of the tumor. Maximal standard uptake value (SUVmax) of the tumor was 10.82.

A 76‐year‐old man with a history of prostate cancer was referred to the respirology department of our hospital with nodules in the right upper lobe of the lung on chest computed tomography (CT). He had a smoking history of 20 pack‐years, and no exposure to any environmental fumes or dust. His physical examination was unremarkable. Laboratory findings were within normal limits. The chest CT revealed a 10.5 x 9.7 mm nodule (Figure 1a) at the peripheral part of B2b, close to V3aii and a 7.8 x 5.4 mm nodule in S2 (Figure 1b) located in the right upper lobe. We assessed these findings as not typical of lung cancer, and decided to undergo radiological follow‐up. One month later, the larger nodule had shrunk to 8.1 x 8.4 mm (Figure 1c). CT follow‐up was continued until 4 months after the first visit, when both nodules had shrunk to an almost unrecognizable size (Figure 1e,f). From this clinical course, the nodules were diagnosed as nonmalignant and follow‐up was stopped. Two years later, that patient was again referred to our hospital with a nodule in the right upper lobe of the lung. CT revealed a 19.3 x 10.4 mm nodule at the peripheral part of B2b in exactly the same place as the larger nodule revealed 2 years previously, close to V3aii (Figure 1g). We evaluated that the larger nodule had enlarged again. The smaller nodule had completely vanished. Maximal standard uptake value (SUVmax) of positron emission tomography/computed tomography (PET/CT) was 10.82 (Figure 2). Bronchoscopy was performed and following transbronchial fine needle aspiration the tumor was diagnosed as squamous cell carcinoma. Surgical resection was scheduled and right upper lobectomy and ND2a‐1 lymph node dissection was performed.

**FIGURE 3:**
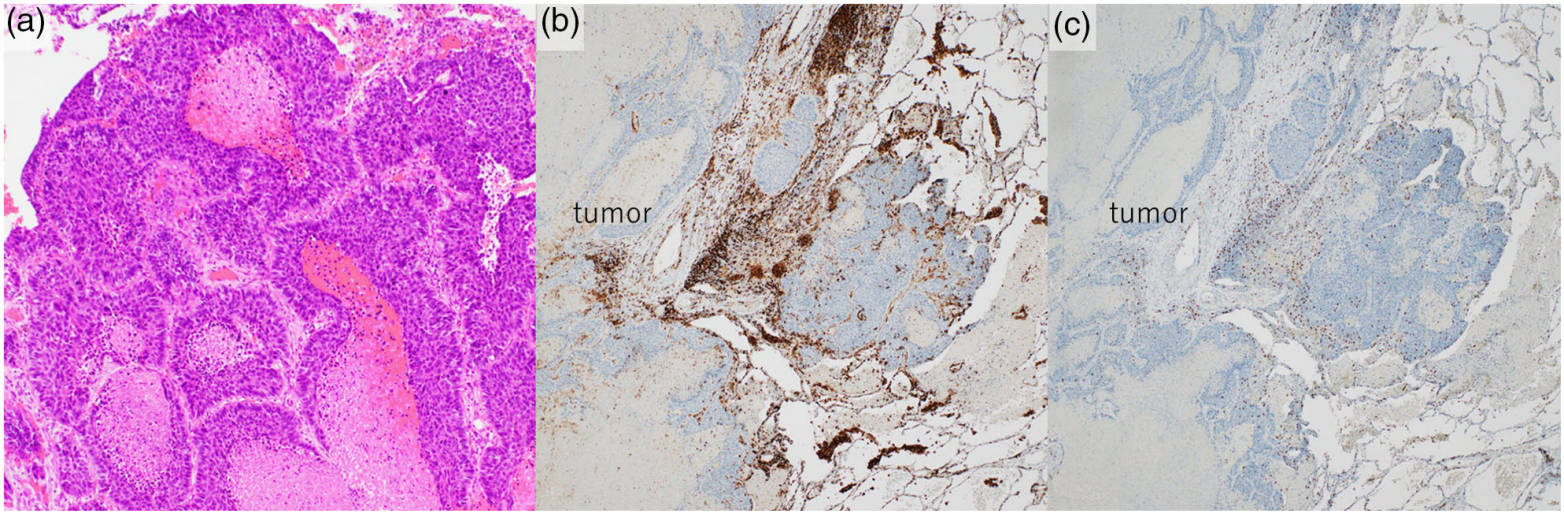
Pathological findings. (a) Hematoxylin and eosin (H&E) stain: Sheets of basaloid cells showed a peripheral palisading pattern. Widespread necrosis was seen inside the tumor. (b) CD4: CD4‐positive lymphocytes infiltrated around the tumor and interstitial space. (c) CD8: CD8‐positive lymphocytes infiltrated around the tumor and interstitial space.

Pathological examination revealed sheets of hyperchromatic basaloid cells with minimal cytoplasm and peripheral palisading with alveolar structure. Widespread necrosis was seen inside the tumor (Figure 3a). Immunostaining showed that the tumor cells were positive for p40 and the tumor was diagnosed as basaloid squamous cell carcinoma. In addition, immunostaining showed infiltration of CD4‐positive lymphocytes and CD8‐positive lymphocytes around the tumor and the interstitial space (Figure 3b,c).

## DISCUSSION

SR of the non‐small cell lung cancer is relatively rare and only 25 cases have previously been reported.^2^ Most patients are of an advanced age, median age of 69 years. SR of basaloid squamous cell carcinoma has not previously been reported, and to the best of our knowledge this is the first report.

The exact mechanisms of cancer SR remain unknown, but several theories such as immunoregulation after infection or injury, interactions between neuropsychological and immunological systems, hormonal mechanisms, and normalization of cell differentiation have been reported.^3^ Most of the SR cancer reports consider the mechanism to be immunological,^3^, ^4^ and SR of tumors which are recognized for their immunogenicity such as melanoma, renal cell carcinoma and hematological cancers is relatively frequent.^2^

Basaloid squamous cell carcinoma of the lung is a rare subtype of lung squamous cell carcinoma. It is an uncommon histological variant of lung cancer composed of cells exhibiting cytological and tissue architectural features of both squamous cell lung carcinoma and basal cell carcinoma, while the proportion of squamous cell components is less than 50%.^5^ It has previously been reported that basaloid squamous cell carcinoma accounts for 3.9%–5.2% of all lung squamous carcinomas, and typically show rapid clinical progression, rapid growth rate, and a poorer prognosis compared to conventional squamous cell carcinoma of the lung.^5^, ^6^ Li et al. performed the molecular characteristic comparison of basaloid squamous cell carcinoma and common squamous cell carcinoma of the esophageal, and reported that basaloid squamous cell carcinoma showed downregulation of CCL21 which associated with immune response.^7^ They also reported a worse response of immune‐chemotherapy in basaloid squamous cell carcinoma.^7^ This poor immune response tendency might be similar in the same subtype of lung cancer, and might be the reason for the rarity of SR in basaloid squamous cell carcinoma.

In the present case, pathological investigation revealed CD4‐positive lymphocytes and CD8‐positive lymphocytes around the tumor and interstitial space. Similar to our case, lymphocyte infiltration around the tumor was found in the resected lung cancer specimen in patients undergoing SR in other studies.^8^, ^9^ Haruki et al., and Moriyama et al. reported CD8‐positive lymphocyte infiltration in lung cancer specimens which spontaneously regressed.^8^, ^9^ These reports and our case suggest that CD‐8 positive and CD‐4 positive lymphocytes might play an important role in SR of cancer.

In conclusion, in the present study, we report a very rare case of SR of basaloid squamous cell carcinoma of the lung. Stimulation of the immune system is considered to be the cause of the SR, and CD‐8 positive and CD‐4 positive lymphocytes. Since the tumor in the case reported in the present study enlarged after follow‐up stopped due to SR, we should be careful in determining when to stop follow‐up, even if the tumor has shrunk or disappeared.

## AUTHOR CONTRIBUTIONS

Conception and design: Koike S; Data analysis and interpritaion: Koike S; Manuscript writing: Koike S; and final approval of manuscript: all authors.
